# Anti-CD22/CD20 Bispecific Antibody with Enhanced Trogocytosis for Treatment of Lupus

**DOI:** 10.1371/journal.pone.0098315

**Published:** 2014-05-19

**Authors:** Edmund A. Rossi, Chien-Hsing Chang, David M. Goldenberg

**Affiliations:** 1 Immunomedics, Inc., Morris Plains, New Jersey, United States of America; 2 IBC Pharmaceuticals, Inc., Morris Plains, New Jersey, United States of America; 3 Center for Molecular Medicine and Immunology, Morris Plains, New Jersey, United States of America; Carl-Gustav Carus Technical University-Dresden, Germany

## Abstract

The humanized anti-CD22 antibody, epratuzumab, has demonstrated therapeutic activity in clinical trials of lymphoma, leukemia and autoimmune diseases, treating currently over 1500 cases of non-Hodgkin lymphoma, acute lymphoblastic leukemias, Waldenström’s macroglobulinemia, Sjögren’s syndrome, and systemic lupus erythematosus. Because epratuzumab reduces on average only 35% of circulating B cells in patients, and has minimal antibody-dependent cellular cytotoxicity and negligible complement-dependent cytotoxicity when evaluated *in vitro*, its therapeutic activity may not result completely from B-cell depletion. We reported recently that epratuzumab mediates Fc/FcR-dependent membrane transfer from B cells to effector cells via trogocytosis, resulting in a substantial reduction of multiple BCR modulators, including CD22, CD19, CD21, and CD79b, as well as key cell adhesion molecules, including CD44, CD62L, and β7 integrin, on the surface of B cells in peripheral blood mononuclear cells obtained from normal donors or SLE patients. Rituximab has clinical activity in lupus, but failed to achieve primary endpoints in a Phase III trial. This is the first study of trogocytosis mediated by bispecific antibodies targeting neighboring cell-surface proteins, CD22, CD20, and CD19, as demonstrated by flow cytometry and immunofluorescence microscopy. We show that, compared to epratuzumab, a bispecific hexavalent antibody comprising epratuzumab and veltuzumab (humanized anti-CD20 mAb) exhibits enhanced trogocytosis resulting in major reductions in B-cell surface levels of CD19, CD20, CD21, CD22, CD79b, CD44, CD62L and β7-integrin, and with considerably less immunocompromising B-cell depletion that would result with anti-CD20 mAbs such as veltuzumab or rituximab, given either alone or in combination with epratuzumab. A CD22/CD19 bispecific hexavalent antibody, which exhibited enhanced trogocytosis of some antigens and minimal B-cell depletion, may also be therapeutically useful. The bispecific antibody is a candidate for improved treatment of lupus and other autoimmune diseases, offering advantages over administration of the two parental antibodies in combination.

## Introduction

Although the previous view of B cells in autoimmunity was as precursors of deleterious autoantibody-producing plasma cells, they have more recently been ascribed other roles in the pathogenesis of autoimmune diseases, including systemic lupus erythematosus (SLE or lupus), such as cytokine production, presentation of autoantigens, promotion of breakdown of T-cell tolerance, and possibly activation of populations of T cells with low affinity toward autoantigens [1]–[3]. Due to the central role of B cells in the pathogenesis of autoimmunity, targeted anti-B-cell immunotherapies should offer therapeutic opportunities in the treatment of SLE. Of note, belimumab, which was approved recently for the treatment of SLE, is a mAb that inhibits activation of B cells by blocking B-cell activating factor [4].

CD22, a B-lymphocyte-restricted member of the immunoglobulin superfamily that regulates B-cell activation and interaction with T cells [5]–[17], is yet another attractive target. The humanized mAb, epratuzumab (hLL2 or IMMU-103) [18], [19], has demonstrated therapeutic activity in clinical trials of lymphoma and autoimmune disease, having treated over 1500 cases of non-Hodgkin lymphoma (NHL) [1], [20]–[25], acute lymphoblastic leukemias [26], Sjögren’s syndrome [27], and SLE [28]–[31]. Although epratuzumab has indicated clinical activity [1], [20]–[31], its mechanism of action (MOA) remains obscure. Because epratuzumab has modest antibody–dependent cellular cytotoxicity (ADCC) and negligible complement-dependent cytotoxicity (CDC) *in vitro* [5], [6], we postulated that, unlike CD20-targeting mAbs, such as rituximab, its therapeutic action may not result from its moderate depletion of circulating B cells.

Recently, we identified trogocytosis as a previously unknown, and potentially important, MOA of epratuzumab, which may be pertinent to its therapeutic effects in B-cell-regulated autoimmune disease [32]. Trogocytosis [33], also referred to as shaving [34], is a mechanism of intercellular communication [35]–[38] where two different types of cells initially form an immunological synapse due to the interaction of receptors and ligands on acceptor and donor cells, respectively [39]–[41], after which the ligands and portions of the associated donor cell membrane are taken up and subsequently internalized by the acceptor cell. Importantly, trogocytosis may regulate immune responsiveness to disease-associated antigens and can either stimulate or suppress the immune response [39]. In studies with an *ex-vivo* model, we demonstrated that epratuzumab mediated a significant reduction of the B-cell surface levels of key B-cell antigen receptor (BCR) signal-modulating proteins, including CD22, CD19, CD21 and CD79b, and also important cell-adhesion molecules, such as CD44, CD62L and β7-integrin, that are involved in B-cell homeostasis, activation, recirculation, migration, and homing. The reduction of the surface proteins on B cells occurred via trogocytosis to FcγR-bearing effector cells, including monocytes, granulocytes and NK cells [32]. Importantly, we verified that these key proteins were reduced significantly on B cells of SLE patients receiving epratuzumab therapy, compared to treatment-naïve patients. We proposed that epratuzumab-mediated loss of BCR modulators and cell-adhesion molecules incapacitates B cells, rendering them unresponsive to activation by T-cell-dependent antigens, leading to therapeutic control in B-cell-mediated autoimmune disease [32].

The primary MOA of anti-CD20 mAbs in NHL and autoimmune disease is B-cell depletion. Whereas elimination of healthy B cells is likely unavoidable for effective therapy of NHL, it may be detrimental in the therapy of autoimmune diseases due to the increased susceptibility to serious, possibly life-threatening, infections. Although rituximab was approved in 2006 for rheumatoid arthritis [42], it failed to achieve the primary endpoint in the LUNAR trial of SLE [43], despite encouraging prior results. Moreover, an analysis of efficacy and safety data from BELONG, a phase III trial of ocrelizumab (humanized anti-CD20), found that the treatment did not significantly improve renal response rates compared with treatment controls, and was associated with a higher rate of serious infections [44]. In both trials, the anti-CD20 mAbs achieved numerically, but not statistically, better responses than the control group, which received standard lupus therapies including steroids, in part because many patients were unable to complete the designed regimen due to serious infections resulting from B-cell depletion. In fact, BELONG was terminated early because of this.

Since both CD20 and CD22 targets have shown activity with their respective antibodies given to patients with autoimmune disease, we postulated that a bispecific antibody (bsAb) targeting both antigens could have superior properties to either parental mAb alone or even a combination of both. Herein, we describe for the first time enhanced trogocytosis mediated by bispecific antibodies targeting neighboring cell-surface proteins. We have developed an anti-CD22/CD20 bispecific hexavalent antibody (bsHexAb), 22*-(20)-(20), that combines the advantages of both anti-CD20 and anti-CD22 therapies, with enhanced trogocytosis and reduced B-cell depletion, compared to the parental anti-CD22 and anti-CD20 mAbs, respectively. This bsAb, which was shown previously to have favorable pharmacokinetics and *in vivo* stability [45], could be highly effective in the therapy of autoimmune diseases, including SLE.

## Methods

### Antibodies, Cell Lines and Reagents

**Figure 1:**
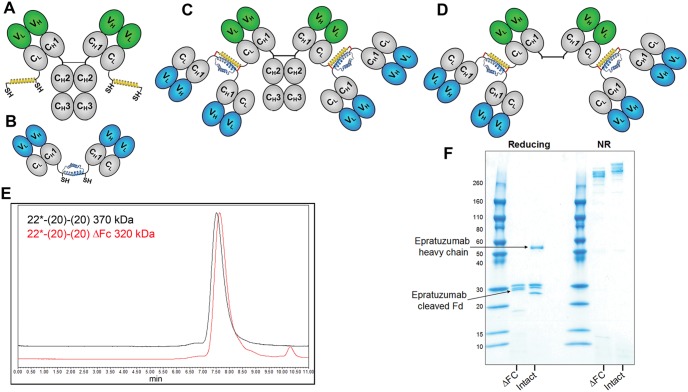
DNL modules and bsHexAb structures. (**A**) C_k_-AD2-IgG-epratuzumab, an IgG-AD2 module with an AD2 fused to the carboxyl-terminal end of each kappa light chain. (**B**). Dimeric C_H_1-DDD2-Fab-veltuzumab, or C_H_1-DDD2-Fab-hA9, Fab-DDD modules with DDD2 fused to the carboxyl-terminal end of the F_d_ chain (**C**). Structure of 22*-(20)-(20) or 22*-(19)-(19), bsHexAbs comprising C_k_-AD2-IgG-epratuzumab and two dimeric C_H_1-DDD2-Fab-veltuzumab or C_H_1-DDD2-Fab-hA19 modules, respectively. (**D**) Structure of 22*-(20)-(20) with the Fc removed. Variable (V, blue or green) and constant (C, grey) domains of IgG heavy (H) and light (L) chains are represented as ovals. The DDD2 (dimerization and docking domain) and AD2 (anchor domain) peptides are shown as blue and yellow helices, respectively, with the locations indicated for the reactive sulfhydryl groups (SH) and the “locking” disulfide bridges indicated as red lines. (**E**) SE-HPLC showing the homogeneity of 22*-(20)-(20) and the expected small shift in retention following removal of the Fc, which comprises 13% of the protein. (**F**) Reducing (left) and non-reducing (right) SDS-PAGE showing the elimination of the intact epratuzumab heavy chain (intact lane) and the appearance of the resulting cleaved epratuzumab Fd following removal of the Fc (ΔFc lane).

Epratuzumab (humanized anti-CD22 IgG1κ), veltuzumab (humanized anti-CD20 IgG1κ) [46], labetuzumab (humanized anti-CEACAM5 IgG1κ) [47], and hA19 (humanized anti-CD19 IgG1κ) were provided by Immunomedics, Inc. Rituximab was obtained from a commercial source. The Fc fragment was removed from rituximab and 22*-(20)-(20) by digestion with pepsin at pH 4.0 (Figure 1). Daudi and Raji human Burkitt lymphoma cell lines were from ATCC (Manassas, VA). All cell lines, PBMCs and isolated blood cells were maintained in RPMI 1640 media (Life Technologies, Inc., Gaithersburg, MD), supplemented with 10% heat inactivated fetal bovine serum (Hyclone, Logan, UT).

### Construction of bsHexAbs

The construction of 22*-(20)-(20) using the Dock-and-Lock (DNL™) method, and its biochemical characterization, have been described previously [45]. The 22*-(19)-(19) was assembled using the same method. Independent stable transfectant SpESFX-10 myeloma cell lines [48] produced C_k_-AD2-IgG-epratuzumab (Figure 1A) and dimeric C_H_3-DDD2-Fab modules of veltuzumab and hA19 (Figure 1B), which were isolated from culture broths by affinity chromatography using MAb-Select and Ni-Sepharose (GE Healthcare) resins. C_k_-AD2-IgG-epratuzumab was combined with 2.1 mole equivalents (10% excess) of C_H_3-DDD2-Fab-veltuzumab or C_H_3-DDD2-Fab-hA19 to generate 22*-(20)-(20) or 22*-(19)-(19), respectively (Figure 1C). DNL conjugations were accomplished by overnight room temperature incubation of the mixtures with 1 mM reduced glutathione, followed by the addition of 2 mM oxidized glutathione. Homogeneous preparations of the bsHexAbs were purified from the reaction mixture with MAb-Select affinity chromatography (Figure 1E and F).

### Ethical Approval

Because blood fractions from anonymous donors were purchased from a commercial source, and no animals were used, this study is not governed by the Declaration of Helsinki, and, consent and approval from an ethical committee were not required.

### Preparation of Blood Cell Fractions

Heparinized whole blood (buffy coat) from anonymous healthy donors was purchased from The Blood Center of New Jersey (East Orange, NJ). PBMCs were isolated by density gradient centrifugation on UNI-SEP tubes (Novamed Ltd., Jerusalem, Israel). Depletion of NK cells and isolation of monocytes from PBMCs was accomplished using MACS separation technology (Miltenyi Biotec, Auburn, CA) with human anti-CD56 and anti-CD14 microbeads, respectively, according to the manufacturer’s recommended protocol.

### *Ex vivo* Experiments

For trogocytosis experiments, PBMCs (1.5×10^6^ cells/mL) were treated in triplicate with 10 µg/mL mAbs or bsHexAbs overnight (16–18 h) at 37°C in non-tissue culture treated 48-well plates, before analysis by flow cytometry. For each antigen evaluated, incubation with the isotype control labetuzumab (anti-CEACAM5, irrelvant mAb) resulted in fluorescence staining that was indistinguishable from untreated cells. Surface antigen levels, shown as % of control, were obtained by dividing the mean fluorescent intensity (MFI) of the cells treated with a test agent by that of the cells treated under the same conditions with labetuzumab, and multiplying the quotient by 100.

For studying B-cell depletion, PBMCs were incubated for two days, before addition of anti-CD19-PE, anti-CD79b-APC, 7-AAD, and 30,000 CountBright Absolute Counting Beads (Life Technologies) to each tube. For each sample, 8,000 CountBright beads were counted as a normalized reference.

For CDC, cells were seeded in black 96-well plates (Nunc) at 5×10^4^ cells in 50 µL/well and incubated with serial dilutions of test and control mAbs in the presence of human complement (1∶20 final dilution, Quidel Corp.) for 2 h at 37°C and 5% CO_2_. Viable cells were then quantified using the Vybrant Cell Metabolic Assay Resazurin kit (Invitrogen). Controls included cells treated with 0.25% Triton X-100 (100% lysis) and cells treated with complement alone (background).

For ADCC, target cells were incubated with each test article in triplicate for 30 min at 37°C and 5% CO_2_. Freshly isolated PBMCs were then added at a predetermined optimal effector to target ratio of 50∶1. After a 4-h incubation, cell lysis was assessed by CytoTox-One (Promega).

Student’s t-test was used to evaluate statistical significance (*P*<.05).

### Flow Cytometry

**Figure 2:**
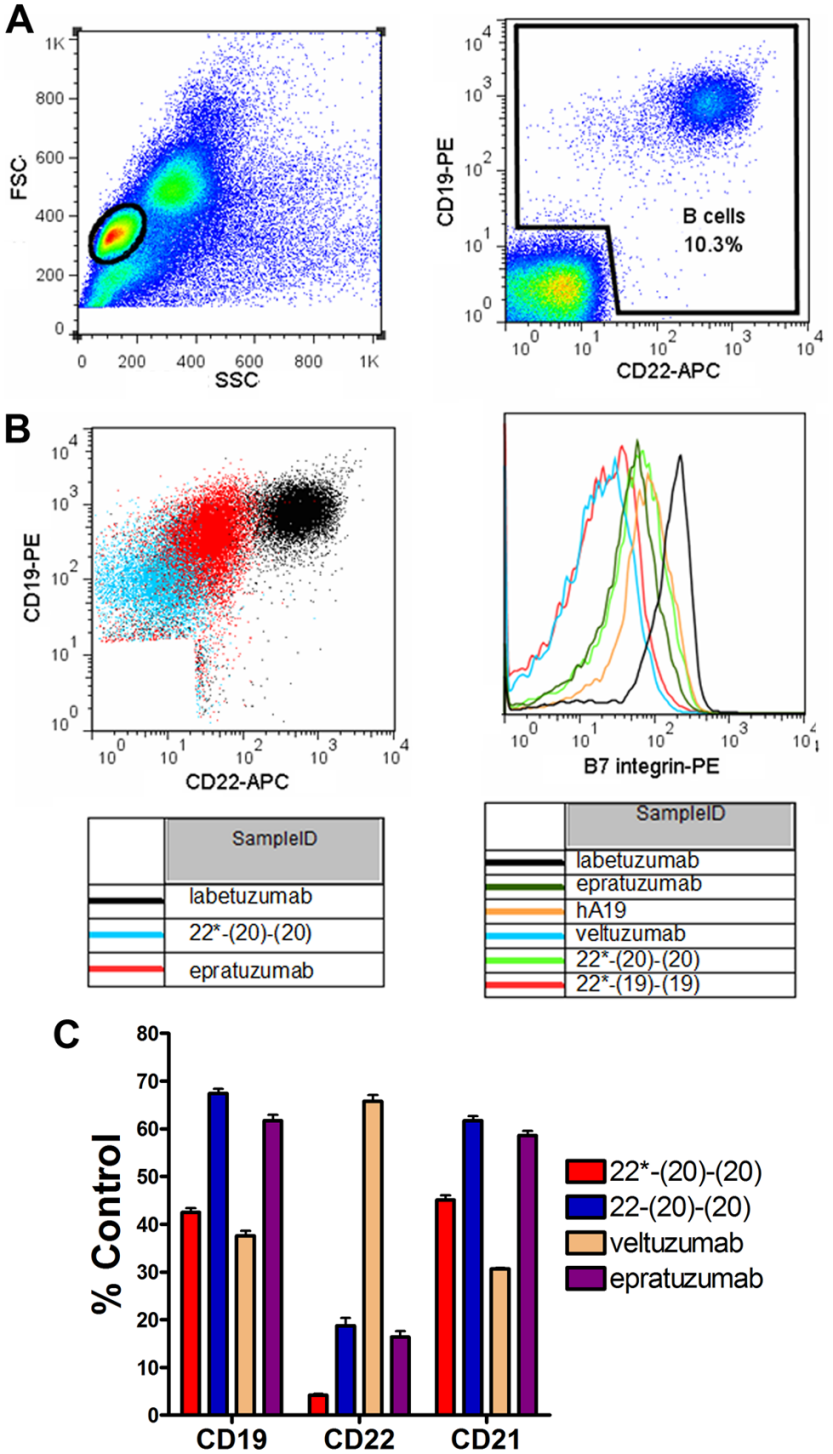
Analysis of trogocytosis by flow cytometry. PBMCs were incubated overnight with 10 µg/mL of various mAbs or bsHexAbs prior to measurement of surface antigens by flow cytometry. (**A**) Gating of lymphocytes by forward vs. side scattering (Left) and B cells from the lymphocyte gate using CD19 and CD22 staining (Right) following treatment with control mAb (labetuzumab). (**B**) Example dot-plots comparing CD19 and CD22 staining on B cells following treatment of PBMCs with 22*-(20)-(20), epratuzumab and labetuzumab (Left) and histograms showing β7 integrin staining following treatment with the indicated mAbs or bsHexAbs (Right). (**C**) Trogocytosis mediated by C_k_ and C_H_3-based bsAbs. PBMCs were incubated overnight with 10 µg/mL 22*-(20)-(20), 22-(20)-(20), veltuzumab, epratuzumab or labetuzumab (control), prior to measurement of surface CD19, CD22 and CD21 by flow cytometry. Results are shown as the % MFI of the control treatment. Error bars, Std. Dev.

Cell mixtures were stained in a one-step procedure by incubating with mixed flourochrome-antibody cocktails in 1% BSA-PBS for 30 min at 4°C. Following staining, cells were washed twice with 1% BSA-PBS and samples were acquired on a FACSCalibur flow cytometer (Becton Dickinson, Franklin Lakes, NJ). For multi-color acquisition, compensation adjustments were performed using single color samples. The same instrument settings were maintained in acquiring all samples. Data were analyzed with Flowjo software (version 7.6.5, Treestar Inc., Ashland, OR). Lymphocytes were gated by forward and side scattering. B cells were identified from the lymphocyte gate using two B-cell specific markers (CD19, CD20, CD22 or CD79b), depending on the specific antibody used for treatment, in order to avoid missing any cells where treatment reduced one marker to near background levels (Figure 2A).

### Fluorochrome-antibody Conjugates Used with Flow Cytometry

The following fluorochrome-anti-human mAbs were used according to the manufacturer’s recommendations. Anti-CD22 (FITC and APC, clone HIB22), anti-CD21 (FITC, clone LT21), anti-CD79b (APC and PE, clone CD3-1), and anti-CD19 (PE/Cy7, clone HIB19) were from Biolegend (San Diego, CA). Anti-CD19 (PE and FITC, clone LT19) and anti-CD20 (PE, clone LT20), were from Miltenyi Biotec. Anti-CD44 (FITC, clone L178), anti-β7 integrin (PE, clone FIB504), and anti-CD62L (FITC, clone DREG-56) were from BD Biosciences (San Jose, CA). Binding specificity was confirmed using isotype control mAbs. For exclusion of dead cells, 7-AAD (Life Technologies) was added prior to flow cytometry analysis. Preincubation of PBMCs or Daudi cells with epratuzumab or 22*-(20)-(20) at 4°C did not inhibit detection of CD22, CD19, CD21, or CD79b with anti-CD22 clone HIB22, anti-CD19 clone HIB19, anti-CD21 clone LT21, or anti-CD79b clone CD3-1, respectively. Preincubation with rituximab, veltuzumab, or 22*-(20)-(20) blocked detection of CD20 with anti-CD20 clone LT20. Preincubation with hA19 (humanized anti-CD19) or 22*-(19)-(19) blocked detection of CD19 with anti-CD19 clone LT19 (as well as 11 additional anti-CD19 mAbs).

### Fluorescence Microscopy

Monocytes were purified from freshly isolated PBMCs by positive selection and their plasma membranes were labeled with the PKH26-Red fluorescent cell labeling kit (Sigma, St. Louis, MO), following the manufacturer’s recommended procedure. Daudi cell plasma membranes were labeled with the PKH67-Green fluorescent cell labeling kit (Sigma). Fluorescent-labeled monocytes and Daudi cells were mixed 2∶1 (7.5×10^6^/mL total cell density) and incubated at room temperature for 30 minutes in the presence of 10 µg/mL 22*-(20)-(20) or labetuzumab.

## Results

### Trogocytosis

The 22*-(20)-(20) bsHexAb (Figure 1C) exhibited the broadest and most extensive trogocytosis, reducing each of CD22, CD20, CD19, CD21, CD79b, CD44, CD62L, and β7-integrin more than epratuzumab, and to a similar extent as veltuzumab, except for CD22, which was reduced much more with the 22*-(20)-(20) (Table 1). The gating strategy (Figure 2A), example dot-plots (Figure 2B, left panel) and histograms (Figure 2B, right panel) demonstrating trogocytosis are shown in Figure 2. In general, 22*-(19)-(19) showed intermediate trogocytosis, with less antigen reduction than 22*-(20)-(20), but more than epratuzumab for select antigens, such as CD21 (*P* = .0173) and presumably CD19. We were unable to measure CD19 levels following treatment of PBMCs with hA19 or 22*-(19)-(19), because these antibodies block detection of the antigen (12 commercial CD19 mAbs tested). However, the considerable reduction of CD21 suggests a similar reduction of CD19. Similarly, CD20 detection was blocked with veltuzumab or 22*-(20)-(20), which each presumably remove most of the CD20 from B cells. The 22*-(20)-(20) mediated significantly (*P*<.001) more trogocytosis compared to 22-(20)-(20), which is a bsHexAb where the additional veltuzumab Fabs are fused at the end of the heavy chain, instead of at the end of the light chain (Figure 2C) [45], [49].

**Table 1:** Percent reduction of B-cells antigens following overnight treatment of PBMCs.

Treatment	CD22	CD20	CD19	CD21	CD79b	CD62L	CD44	β7-Int
22*-(20)-(20)	96.2 (±1.3)^a^	n.d.	83.9 (±6.2)^c^	77.7 (±0.3)^c^	61.7 (±7.9)^a^	81.3 (±11.3)^d^	51.0 (±6.5)^d^	81.0 (±1.6)^d^
22*-(19)-(19)	93.7 (±1.6)^b^	25.3 (±5.4)^c^	n.d.	73.5 (±4.5)^c^	42.0 (±6.7)	64.5 (±7.0)	30.45 (±5.2)	57.7 (±3.5)
**Epratuzumab**	92.5 (±2.1)^b^	11.8 (±1.7)	56.1 (±6.0)	59.1 (±6.4)	39.4 (±4.5)	65.3 (±8.0)	31.3 (±3.9)	59.1 (±5.7)
**Veltuzumab**	58.5 (±6.2)	n.d.	92.9 (±2.3)^a^	84.8 (±0.5)^a^	45.0 (±10.3)	77.5 (±7.2)^e^	59.2 (±5.4)^e^	83.1 (±2.1)^e^
**hA19**	29.0 (±3.4)	17.3 (±8.2)	n.d.	68.0 (±3.1)^c^	32.8 (±7.6)	52.6 (±5.8)	31.9 (±4.6)	42.8 (±3.4)

Average % reduction from three experiments using PBMCs form independent donors. n.d., not measured due to blocked detection by the specific treatment. Significantly (*P*<0.05) more reduction than:

a all other agents;

b veltuzumab and hA19;

c epratuzumab;

d all but veltuzumab.

e Not significantly different from 22*-(20)-(20).

**Figure 3:**
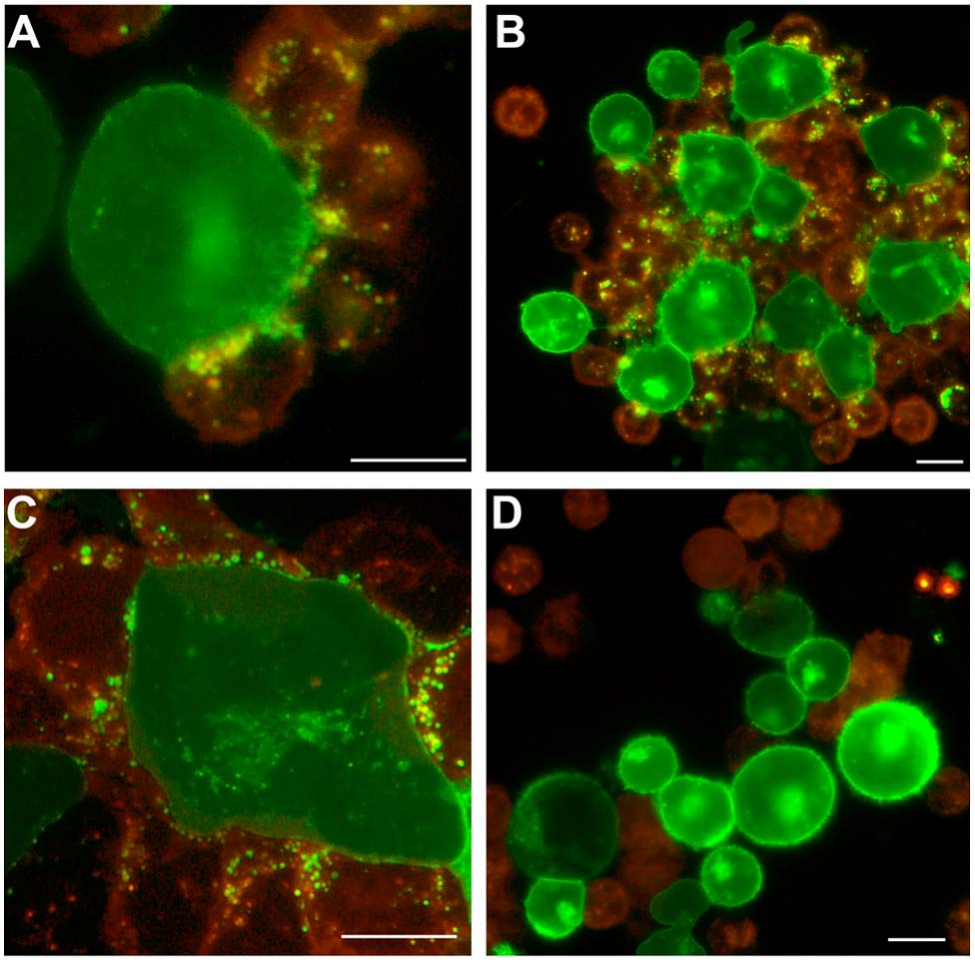
Fluorescence microscopy showing trogocytosis induced with 22*-(20)-(20). Purified monocytes labeled with PKH26-Red fluorescence were mixed 2∶1 with Daudi cells labeled with PKH67-Green fluorescence, and treated with 22*-(20)-(20) (**A–C**) or labetuzumab (**D**) at 10 µg/mL. Fluorescent images were captured after 30 min at room temperature with an Olympus BX66 microscope (Shinjuko, Tokyo, Japan) equipped with a Mercury-100W laser (Chiu Technical Corp., Kings Park, NY), using an Olympus 40X/0.75 air objective lens and a Kodak DC290 Camera (Rochester, New York) set at 115X zoom. A WB filter was used to allow simultaneous fluorescence of both red and green fluorochromes. Images were captured and processed using Adobe Photoshop CS3 v.10 software with a Kodak Microscopy Documentation System 290 plug-in application. Bars: 10 µm.

The flow cytometry results demonstrating trogocytosis were further supported by fluorescence microscopy studies (Figure 3). Purified monocytes and Daudi (B-cell NHL) cells were membrane-labeled with red and green fluorochromes, respectively, and combined. Similar to what was observed previously with epratuzumab [32], addition of 22*-(20)-(20) to the cell mixture resulted in the rapid formation of immunological synapses and cell clustering between Daudi cells and monocytes, and subsequent trogocytosis of green Daudi membrane components to the red-stained monocytes (Figure 3A–C). Addition of the control mAb did not result in any evident trogocytosis, even where Daudi cells and monocytes were juxtaposed (Figure 3D).

### B-cell Depletion

**Figure 4:**
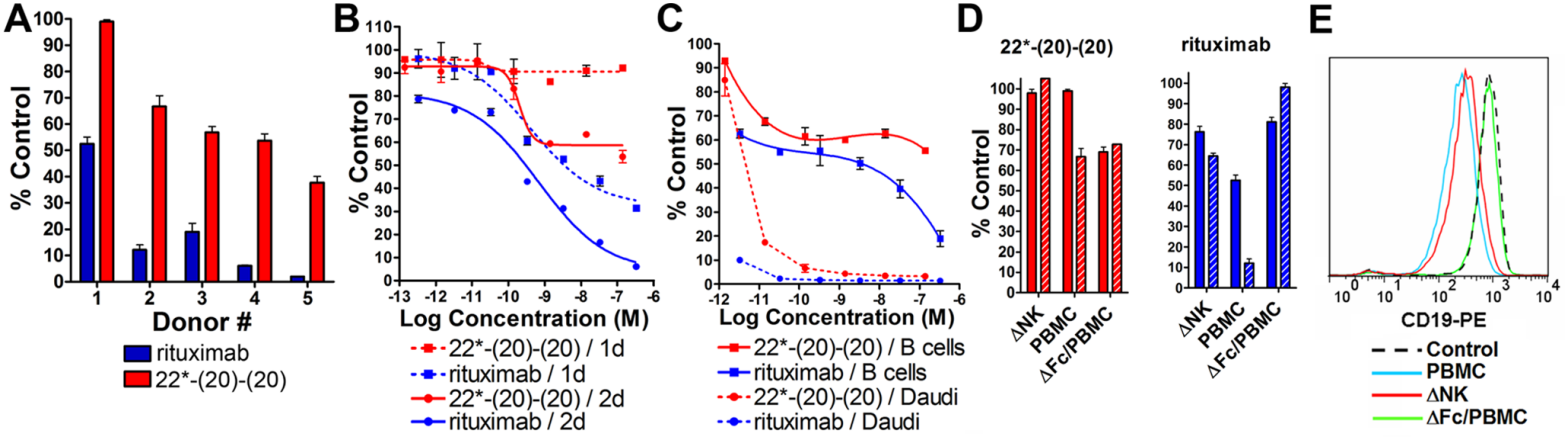
B-cell depletion. Freshly isolated PBMCs were incubated for two days with 22*-(20)-(20) (red) or rituximab (blue) prior to counting the viable B cells The relative viable B cell count is expressed as % Control, which was derived by dividing the specific B cell count by that measured following treatment with the control mAb (labetuzumab). Error bars, Std. Dev. (**A**) B-cell depletion with 140 nM rituximab or 22*-(20)-(20) in PBMCs from 6 unique donors. (**B**) B-cell depletion at 24 h and 48 h with antibody titrations using PBMCs from Donor 4. (**C**) Daudi Burkitt lymphoma cells were spiked in PBMCs from Donor 3 and treated with titrations of the antibodies. Daudi and normal B cells were separated by forward scattering and counted independently. (**D**) 140 nM of 22*-(20)-(20) (left, red) or rituximab (right, blue) were incubated with NK-depleted (ΔNK) or intact PBMCs, which were alternatively treated with Fc-deleted fragments (ΔFc/PBMC) of each antibody. Donor 1, solid bar; Donor 2, hatched bar. (**E**) Reduction of CD19 on B cells by trogocytosis. Control was PBMCs treated with labetuzumab (black dashed trace). Fc-deleted 22*-(20)-(20) was incubated with PBMCs (green trace). Intact 22*-(20)-(20) was incubated with PBMCs (blue trace) or NK cell-depleted PBMCs (red trace). Histograms show the fluorescence intensity for anti-CD19-PE.

Treatment of PBMCs under the standard experimental conditions used for trogocytosis (10 µg/mL overnight) with either epratuzumab, hA19, or 22*-(19)-(19) caused minimal (<10%) B-cell depletion (not shown). The B-cell depletion caused by 22*-(20)-(20), specifically as compared to rituximab, was examined with PBMCs from multiple donors, which were treated at various concentrations for two days before counting viable B cells. The maximal level of B-cell depletion varied widely among donors, and for each donor, 22*-(20)-(20) (0–60% depletion) killed significantly (*P*<.0001) fewer B cells compared to rituximab (50–98% depletion) (Figure 4A). As shown using one of the more potent PBMCs (Donor 4), rituximab acted rapidly with considerable depletion after 24 h, whereas 22*-(20)-(20) did induce appreciable depletion at this time point; however, at higher concentrations of the bsHexAb (>1 nM), significant killing (40%) was evident after 2 days (Figure 4B). Both 22*-(20)-(20) and rituximab were considerably more effective at killing Daudi cells, which were spiked into PBMCs, compared to normal B cells (Figure 4C). It is unlikely that CDC is involved, because complement is expected to be removed during PBMC isolation. ADCC, mediated by Fc interactions with NK cells present in the PBMCs, is more likely involved in B-cell depletion. The effect of removal of NK cells (95%) from the PBMCs or deletion of the Fc from the antibodies (Figure 1) was examined using weak (Donor 1) and strong (Donor 2) B-cell-depleting PBMCs (Figure 4D). For rituximab, much less B-cell depletion occurred when NK cells were removed from the PBMCs. It is possible that some ADCC still occurred with residual NK cells or neutrophils that were not eliminated during NK-cell removal and PBMC isolation, respectively. Removal of the Fc from rituximab had an even greater inhibitory effect on B-cell depletion, which was particularly evident with the strong Donor 2. For 22*-(20)-(20), removal of NK cells completely inhibited B-cell depletion with the strong donor. B cells were not depleted from the weak donor, even with intact PBMCs. Unexpectedly, deletion of the Fc from 22*-(20)-(20) did not affect B-cell depletion with the strong donor PBMCs, and markedly increased depletion with the weak donor PBMCs. These results suggest that there are two MOAs of 22*-(20)-(20) engaged in the *ex vivo* assay. ADCC is inhibited by depletion of NK cells. A putative signaling MOA is inhibited by trogocytosis. Removal of the Fc minimizes ADCC and also inhibits trogocytosis, whereas removal of NK cells only reduces ADCC, and not trogocytosis (Figure 4E), which is mostly mediated by monocytes.

### Effector Functions

**Figure 5:**
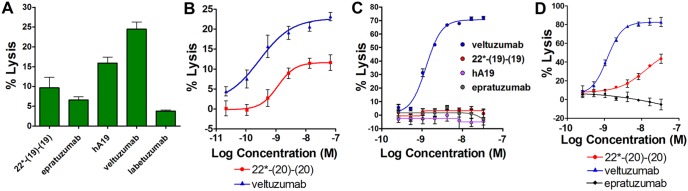
Effector functions. (**A+B**) ADCC. PBMCs were incubated with Daudi cells (50∶1) for 4 h in the presence of the indicated agents at 33 nM (**A**) or with titration (**B**). Similar results were observed with Raji cells and a different PBMC donor. (**C+D**) CDC using Daudi as target cells. Error bars, Std. Dev.

Veltuzumab and rituximab have potent ADCC, whereas hA19 and epratuzumab have moderate and low activity, respectively (Figure 5A). In repeated experiments using different target cell lines and PBMC donors, the bsHexAb 22*-(19)-(19) exhibited significantly lower ADCC than the humanized anti-CD19 mAb, hA19, and the activity was either similar or marginally higher than epratuzumab, depending on the experiment. The ADCC of 22*-(20)-(20) was compared to that of rituximab with titration experiments. Although the level of ADCC varied among donors, rituximab consistently mediated more killing of Daudi cells, with approximately 2-fold greater maximal lysis compared to 22*-(20)-(20) (Figure 5B). Neither epratuzumab, hA19, nor 22*-(19)-(19) mediated CDC *in vitro* (Figure 5C). The CDC of 22*-(20)-(20) was more than 25-fold less potent than veltuzumab (Figure 5D).

## Discussion

B-cell directed mAbs offer promising therapeutic options for SLE as well as other autoimmune diseases. Epratuzumab has shown clinical efficacy with minimal side-effects in SLE [1], [28]–[31], and is in two worldwide Phase III EMBODY™ registration trials (NCT01262365). Rituximab, and possibly other anti-CD20 mAbs, are associated with increased risks of serious infections, due to near wholesale depletion of B cells. The “Black Box Warnings” for rituximab include the reactivation of hepatitis B virus and potentially fatal Progressive Multifocal Leukoencephalopathy (PML), which typically manifests only in individuals with severely compromised immune systems. Clinically, epratuzumab depletes only about 35–45% of circulating B cells and does not increase the risk of infection [28]–[31]. Nonetheless, epratuzumab is effective in SLE and other diseases by mechanisms that remain unclear. Recently, we identified trogocytosis, whereby multiple key proteins, including BCR modulators and adhesion molecules, are stripped from the surface of B cells, as a potentially important MOA of epratuzumab in B-cell regulated autoimmune diseases [32]. We observed that the anti-CD20 mAbs, rituximab and veltuzumab, mediated an even stronger trogocytosis of each antigen (besides CD22). However, the potential of enhanced trogocytosis with anti-CD20 mAbs is diminished, because ultimately the B cells are all killed. Herein, we have identified a novel bsHexAb, 22*-(20)-(20), that mediates a broad and potent trogocytosis of multiple B-cell surface proteins with only moderate B-cell depletion.

An earlier version of an anti-CD22 x anti-CD20 bsHexAb, 22-(20)-(20), which has four Fabs of veltuzumab fused to the Fc of epratuzumab, demonstrated potent killing of lymphoma cell lines *in vitro* [49]. Subsequently, we reported that bsHexAbs of the “Ck” format, with the additional Fabs fused to the end of the light chain, has superior *in vivo* properties, including pharmacokinetics, neonatal FcR binding, and stability, compared to the original format, where Fabs are fused to the end of the heavy chain [45]. Here, we show that the Ck-based 22*-(20)-(20) mediates more trogocytosis compared to the Fc-based 22-(20)-(20). This is likely due to a stronger binding affinity for FcγRs (CD16 and CD64), as was found for FcRn binding.

Trogocytosis with 22*-(20)-(20) reduced the surface levels of CD19, CD21, CD79b, CD44, CD62L, and β7-integrin to similarly low levels as veltuzumab, which were considerably lower than with epratuzumab. Although we were unable to measure the level of CD20 after treatment, it is reasonable to assume that it is reduced to minimal levels, because it is one of the antigens specifically targeted by 22*-(20)-(20) and veltuzumab. Not surprisingly, CD22 is reduced to minimal levels by 22*-(20)-(20), but not with veltuzumab. Trogocytosis, the proposed MOA of epratuzumab, is enhanced with 22*-(20)-(20) by the addition of CD20-binding Fabs to epratuzumab. It is likely that targeting CD20 results in more trogocytosis compared to CD22 targeting, because the former is expressed at a higher level. Another important aspect is that following antibody ligation, CD22, but not CD20, is rapidly internalized [5], [6], which is expected to compete with trogocytosis. Previously, we reported that the Fc-based bsHexAb, 22-(20)-(20), does not internalize rapidly [49], and it is likely that this is also the case for 22*-(20)-(20). The broad and potent trogocytosis mediated by 22*-(20)-(20) may modulate immune B cells more effectively than epratuzumab.

The key advantage of trogocytosis with 22*-(20)-(20) over rituximab or veltuzumab is that the bsHexAb kills less B cells. The extent of B-cell depletion varied considerably using PBMCs from different donors. “Weak” PBMCs had almost no B-cell depletion with 22*-(20)-(20) (50% with rituximab), whereas with “strong” PBMCs, up to 60% of the B cells were depleted with the bsHexAb and nearly 100% were killed with rituximab. Presumably, ADCC is the chief MOA involved in B-cell depletion in the *ex vivo* assay. We have found that *in-vitro* ADCC is highly variable among donors, which likely is responsible for the variability in B-cell depletion. We have observed a correlation between ADCC potency and B-cell depletion with a small number of PBMC specimens that were tested for both activities; however, a systematic study was not performed. Closer inspection of the dose-response curves suggests a biphasic shape, indicating that more than one MOA might be involved in the B-cell killing in the *ex vivo* assays (Fig. 4B and C). Removal of NK cells from the PBMCs, which is expected to eliminate ADCC, completely inhibited B-cell depletion with 22*-(20)-(20). Conversely, removal of the Fc, which eliminates trogocytosis as well as ADCC, resulted in enhanced B-cell depletion. This suggests that the second MOA is a result of the direct action on B cells, and is inhibited by trogocytosis. Previously, we described *in-vitro* cytotoxicity with the Fc-based 22-(20)-(20) on NHL cell lines resulting from signaling mechanisms involving Lyn, Syk, PLCγ2, AKT and NF-κB pathways leading to apoptosis via signaling transduction mechanisms [49], [50]. The Fc-based bsHexAb also caused some *ex-vivo* depletion of B cells [49] even though it has weak ADCC [45], suggesting that normal B-cell death resulted from signaling. The current results indicate that 22*-(20)-(20) also can induce apoptosis of normal B cells. However, stripping the antigens from the cell surface by trogocytosis diminishes the effects of signaling. This does not appear to be the case with rituximab, because removal of its Fc eliminates B-cell depletion. Although CDC is eliminated from the *ex vivo* system, it is likely to play a role *in vivo*. That 22*-(20)-(20) has considerably lower CDC than rituximab could widen the difference in B-cell depletion resulting from immunotherapy with these antibodies.

In this study, we compared two bsHexAbs, each comprising epratuzumab fused at the end of its light chains with four additional Fab fragments to either CD20 or CD19. In general, 22*-(20)-(20) induced more trogocytosis than 22*-(19)-(19), which reduced many of the proteins to a similar extent as epratuzumab. However, CD21, and presumably CD19, were reduced more with 22*-(19)-(19), compared to epratuzumab. Although we believe that 22*-(20)-(20) is a more promising candidate therapeutic for SLE, 22*-(19)-(19), having enhanced trogocytosis of some antigens and minimal B-cell depletion, may also be therapeutically useful.

## Conclusion

The potentially ideal effects that might result from immunotherapy with 22*-(20)-(20), specifically, the extensive reduction via trogocytosis of many key B-cell surface proteins, including CD20, CD22, CD19 and CD21, with only moderate B-cell depletion, cannot be accomplished with a mixture of the two parent mAbs. While a mixture of veltuzumab (or rituximab) and epratuzumab may result in a similarly broad trogocytosis as the bsHexAb, inclusion of the anti-CD20 mAb will cause massive depletion of circulating B cells, rendering SLE patients susceptible to serious infections. Further, infusion of two mAbs, instead of a single agent, would be less convenient for both physicians and patients. Thus, 22*-(20)-(20) may offer an improved next-generation antibody for the therapy of SLE and possibly other autoimmune diseases, without the risk associated with rituximab or other potent anti-CD20 mAbs.
